# Functional Neuroanatomy of the Human Accommodation Response to an “E” Target Varying from -3 to -6 Diopters

**DOI:** 10.3389/fnint.2020.00029

**Published:** 2020-05-21

**Authors:** Xiaoli Lv, Yilei Chen, Wenli Tan, Ying Yu, Hong Zou, Yu Shao, Songhua Zan, Jinhua Tao, Wanhong Miao

**Affiliations:** ^1^Department of Ophthalmology, Shuguang Hospital, Shanghai University of Traditional Chinese Medicine, Shanghai, China; ^2^Department of Ophthalmology, The Second Affiliated Hospital of Zhejiang Chinese Medical University, Hangzhou, China; ^3^Department of Radiology, Shuguang Hospital, Shanghai University of Traditional Chinese Medicine, Shanghai, China

**Keywords:** ocular accommodation, brain, blood oxygenation level-dependent functional magnetic resonance imaging (BOLD-fMRI), stimuli, refractive error

## Abstract

**Background**: We aimed to identify the functional brain networks involved in the regulation of visual accommodation by contrasting the cortical functional areas evoked by foveal fixation to an “E” target, which were subservient to the accommodation responses to a -3/-6 diopter stimulus.

**Methods**: Neural activity was assessed in healthy volunteers by changes in blood oxygen level-dependent (BOLD) signals measured with functional magnetic resonance imaging (fMRI). Twenty-five right-handed subjects viewed the “E” target presented in a hierarchical block design. They participated in two monocular tasks: (i) sustained foveal fixation upon an “E” target on a white background at 33 cm (-3.03D accommodative demand); and (ii) sustained fixation through an attached -3D concave lens (-6D accommodative demand) in front of the fixated eye; each condition cycled through a standard alternating 30-s eye open/30-s eye closed design to provide the BOLD contrast. The total sustained period was 480 s.

**Results**: The contrast between the -3D and the rest condition revealed activation in the occipital lobe (Lingual gyrus, Cuneus, Calcarine_L, and Calcarine_R); cerebellum (Cerebellum_Crus1_L and Cerebellum_6_L); precentral lobe (Precentral_R); frontal lobe (Frontal_Inf_Oper_R and Frontal_Mid_R); and cingulate cortex (Cingulum_Ant_L). With the -3D concave lenses (-6D accommodative demand) in front of the fixated eye, the voxel size and peak intensity of activation in the occipital lobe and cerebellum were greater than with the -3D accommodative demand; emergent activated brain areas included the parietal lobe (bilateral precuneus gyrus and right supramarginal gyrus); the precentral lobe and cingulate cortex failed to reach the threshold in the -6D vs. rest contrast. In the -3D and -6D contrast comparison, the frontal lobe (Frontal_Sup_Medial_L) and parietal lobe (Precuneus_L and Precuneus_R) passed the significance threshold of cluster-level family-wise error (FWE) correction. The mean activation in the -3D and -6D contrast revealed an incremental summation of the activations than that found in the previous -3D vs. rest and -6D vs. rest comparisons.

**Conclusions**: Neural circuits were selectively activated during the -3D/-6D accommodative response to blur cues. Cognitive-perceptual processing is involved in signal regulation of ocular accommodative functions.

## Introduction

In today’s society, the use of digital devices has significantly increased in all age groups, and the visual system may not be able to effectively maintain long periods of overloaded close visually based work, leading to asthenopia. Asthenopia refers to the long-term excessive activity of the visual organs (Long et al., 2017), especially the intraocular and extraocular muscles, such as ciliary muscles, which play a major role in focusing the eyes on close objects (Smith, 1973; Thiagarajan and Ciuffreda, 2013a), and the medial and lateral rectus muscles whose heterotropic movements dominate the convergence and divergence of the eyes (Thiagarajan and Ciuffreda, 2013b). The clinical symptoms of asthenopia usually manifest as an inability to maintain short-distance work, itching, pain around the eyes and orbit, blurred vision, tearing, photophobia, dryness, foreign-body sensation (Sheedy et al., 2003). Many distinct conditions can lead to asthenopia, including accommodative dysfunctions (Jaschinski-Kruza and Schweflinghaus, 1992; García-Muñoz et al., 2014), refractive errors (Kotegawa et al., 2008; Heus et al., 2018), inappropriate lighting conditions (Taylor, 1977; Sliney, 1985; Leśnik and Poborc-Godlewska, 1993), binocular vision anomalies (García-Muñoz et al., 2014). Among these conditions, refractive factors are one of the main causes of asthenopia (Iwasaki et al., 2005). To see objects clearly, patients with refractive errors (such as myopia, hyperopia, astigmatism, etc.) are bound to use more accommodative functions (ciliary muscle contraction) to focus light on the retina.

The complex process of ciliary muscle contraction and its dynamic, accurate and fast functional characteristics, that allow visually focusing on objects at any distance, require precise nervous system control to achieve these fine adjustments in muscle movements (Smith, 1973). When the peripheral ciliary muscles exert more accommodative function, how can cerebral cortex response areas cooperate?

Most of the research on the brain regions that govern ocular accommodative function comes from studies of patients with traumatic brain injury or some sporadic cases (Kawasaki and Borruat, 2005; Green et al., 2010; Rutstein, 2010; Hughes et al., 2017; Hyndman, 2018).There are only a few *in vivo* studies on cerebral activities related to ocular accommodation. In a positron emission tomography (PET) study conducted by Richter et al. (2004), it was revealed that three major brain regions, namely, the cerebellar vermis, the cortices surrounding the right superior temporal sulcus and inferior temporal gyrus, and the extrastriate cortex, were activated when subjects focused on LED stimuli illuminated at a near (24 cm, 4.16 D) vs. a far (3.0 m, 0.33 D) distance. In the above study, the accommodative stimuli only varied with distance from the fixating eye. As we know the accommodative response level varied between stimuli at the different distances and with variations in the concave lenses stimulus level (Thiagarajan and Ciuffreda, 2013a). Thus, additional stimulus paradigms are certainly needed to more precisely understand how cortical responses occur when dioptric changes are made.

The purpose of this study was to investigate the blood oxygen level-dependent (BOLD) response changes to contrasts of stimulus distance (33 cm, −3.03 D) and with the addition of a -3D concave lens (summation for the total stimulus was -6D) in functional magnetic resonance imaging (fMRI) and to understand how response mechanisms in the cerebral cortex changed with the different accommodative stimuli to lay a foundation for the further clinical study of accommodative asthenopia. In our research paradigm, foveal fixation on the distant stimulus was analogous to the normal using state of the eye, and adding the -3D concave lens to the fixation eye resulted in a state in which more accommodative functions were exerted that mainly simulated the conditions including pseudomyopia caused by excessive near-eye use, incorrect correction of refractive errors, and low-degree hyperopia without glasses.

## Materials and Methods

All methods were reviewed by the Shuguang Affiliated Hospital of Shanghai University of Traditional Chinese Medicine Human Research Ethics Committee. All participants signed written informed consent documents approved by the institutional review board at Shuguang Hospital; study procedures were following the Declaration of Helsinki. Data from this study are available from the corresponding author.

### Subjects

Subjects were staff members and students recruited through the ophthalmology clinics at Shuguang Hospital and Shanghai University of Traditional Chinese Medicine.

Twenty-five young, right-handed, healthy participants with normal or corrected-to-normal visual acuity, and right-eye dominance enrolled in this study. Twenty-one subjects were female, and four were male. The subjects’ mean age was 31 years (range: 18–58). Participants were screened for non-MRI compatible implants, and women were screened for current or suspected pregnancy. Before fMRI scanning, subjects underwent a neurological assessment to exclude subjects with a history of traumatic brain injury or seizures, attentional disorder, and mental illness, and an ophthalmology examination was conducted to exclude subjects with amblyopia, strabismus, visual fatigue (by online questionnaire), and binocular vision dysfunction. The subjects were administered the Edinburgh Handedness Inventory to verify right-hand dominance (Oldfield, 1971). We defined the dominant eye for each subject using a hole-in-the-card dominance test (Seijas et al., 2007).The subjects were also told to not drink tea or coffee on the day of scanning.

Outside the scanner, in pre-experimental trials, subjects wore soft contact lenses to achieve 20/20 distance-corrected vision. Inside the scanner, during the experimental trials, subjects were asked to choose the dominant eye to use in the -3D/-6D task while the other eye was fully occluded by medical gauze.

### Task Paradigm

**Figure 1:**
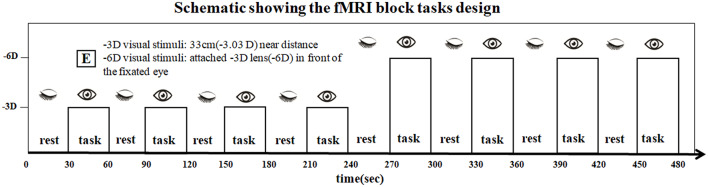
The scan series contained eight visual stimulation blocks containing four blocks with -3D accommodative stimuli and four blocks with -6D accommodative stimuli. The two tasks were presented: (i) sustained foveal fixation upon an “E” target on a white background at 33 cm (-3.03D effective accommodative demand) followed by a rest period with eyes closed, and these conditions alternated sequentially in 30-s epochs; and (ii) the -3D concave lens was attached by the experimenter (-6D effective accommodative demand) in front of the fixated eye, and the eye open and closed conditions alternated in a standard 30-s off/30-s on alternating design to provide the blood oxygen level-dependent (BOLD) contrast. The total sustained period was 480 s.

All subjects used the right eye for viewing. The non-dominant left eye was closed and occluded by gauze. The accommodative “E” stimuli were vertically located at 33 cm (−3.03D), approximately parallel to the visual axis of the right eye. We developed a block design to produce a signal contrast for the -3D/-6D BOLD-fMRI (Figure 1).

The researcher gave instructions to open and close the eye by tapping the subject’s thigh gently. The researchers switched commands every 30 s based on the gestures of a radiological technician. During data processing, brain activation signals from researchers tapping the subject’s thigh would be subtracted without affecting the final analysis of the results.

### Stimuli

The “E” target on a whiteboard was presented at a distance of 33 cm through a mirror on the head coil. The subjects wore a soft contact lens to view the “E” target, which had a height of 2.6 mm, a corresponding angle of view of 0.45°, illumination of approximately 20 cd/m^2^, and 100% contrast. The subjects were asked to look at the central “E” during the task epochs and to try to keep it clear.

-3D accommodative stimulation was achieved by viewing a target at a distance of 33 cm, and -6D accommodative stimulation was achieved by placing a -3D concave lens in front of the dominant eye. The -3D concave lens was fixed on the ending of a bracket about 1 m long. When performing the -6D accommodative task, the researcher placed it in front of the subject’s dominant eye.

### fMRI Data Acquisition

Structural and functional data were obtained using a 3-Tesla SIEMENS MAGNETOM Skyra scanner equipped with a 32-channel head coil tuned to 123.256096 MHz.

The T1 structural images were acquired using a 3D MPRAGE sequence [repetition time (TR) = 2.2 s echo time (TE) = 2.48 ms, flip angle = 8°, matrix size = 256 × 256, 176 slices, voxel size = 0.9 × 0.9 × 1.0 mm^3^]. The superior-inferior field-of-view was 230 mm. The T2 protocol used a turbo spin-echo (TSE) sequence (TR = 4 s TE = 103 ms, flip angle = 150°, matrix size = 384 × 384, 18 slices, voxel size = 0.6 × 0.6 × 6.0 mm^3^). The field-of-view was 220 mm. Functional data were recorded using an echo-planar imaging sequence (TR = 3 s TE = 30 ms, flip angle = 90°, matrix size = 94 × 94, 36 slices, voxel size = 2.0 × 2.0 × 3.0 mm^3^, 160 volumes). The superior-inferior field-of-view was 192 mm.

The subjects were instructed to restrict head motion, and foam padding was used to limit physical movement. All subjects with headphones were placed supine on the gantry with their heads located along the midline of the coil.

### fMRI Data Preprocessing and Analysis

Preprocessing and analyses of the structural and functional fMRI data were performed in SPM12^1^ in a MATLAB 2013b environment^2^. Image analysis was conducted in three steps: (i) preprocessing; (ii) single-subject analysis; and (iii) group analysis.

#### Preprocessing

The functional images for each subject were initially corrected for slice timing. Then, the functional images were realigned to the mean images to account for head motion and coregistered to the T1-weighted anatomical images, which were segmented into gray matter, white matter, cerebrospinal fluid, and other tissues. Then, the deformation field maps were used to normalize all functional images into Montreal Neurological Institute (MNI) space using non-linear registration algorithms. During the normalization, the functional images were resampled with one voxel resolution of 2 × 2 × 2 mm^3^ and spatially smoothed with an 8-mm Gaussian kernel of full-width at half-maximum (FWHM).

#### Single-Subject Analysis

For the statistical analysis of each subject’s fMRI data to assess significant cortical activations, a general linear model (Friston et al., 1995) was derived by convolving a canonical hemodynamic response function with the time series of the experimental stimulus paradigm (-3D/-6D task and rest) to yield a predictor. This predictor, in the next statistical analysis, was tested for its fit with the BOLD signal changes in each voxel of the fMRI data. We regressed six head motion regressors (containing details of translation and rotation realignment parameters) to minimize the effects of head motion in subsequent analyses, set a high-pass filter to remove slow signal drifts with a period longer than 128 s and added time derivatives to the experimental model estimation. After model estimation, individual contrast images were defined for each task (-3D and -6D) and their average or difference between each other to obtain task-related activations for the task (-3D) condition vs. rest or vs. the other (-6D) task condition. This step generated five contrast files for subsequent group analysis.

#### Group Analysis

Activation maps were calculated using a one-sample *t*-test. The five contrast files of each subject were entered into a one-sample *t*-test model for random-effect group analysis. Five group models were created: one activation induced by the -3D accommodative stimulus, the second by the -6D accommodative stimulus, the third by comparing the -3D and -6D conditions, the fourth by comparing the -6D and -3D conditions and the last by the average of the -3D and -6D task conditions.

For all contrasts, we first applied a voxel-level threshold of *P* < 0.001 (cluster-defining threshold); then, all demonstrated brain regions were corrected to *P* ≤ 0.05 at the cluster-level using the family-wise error (FWE) method and random field theory, following the current standard (Woo et al., 2014; Eklund et al., 2016).

## Results

According to T2 images, all subjects were excluded from having organic lesions in the brain. All subjects reported that the tasks were easily implemented. Several participants over 40 years of age noted that the target appeared as a steady blur when the -3D concave lens was placed in front of their eyes.

### Activation in -3D vs. Rest (Eye Closed) Conditions

**Figure 2:**
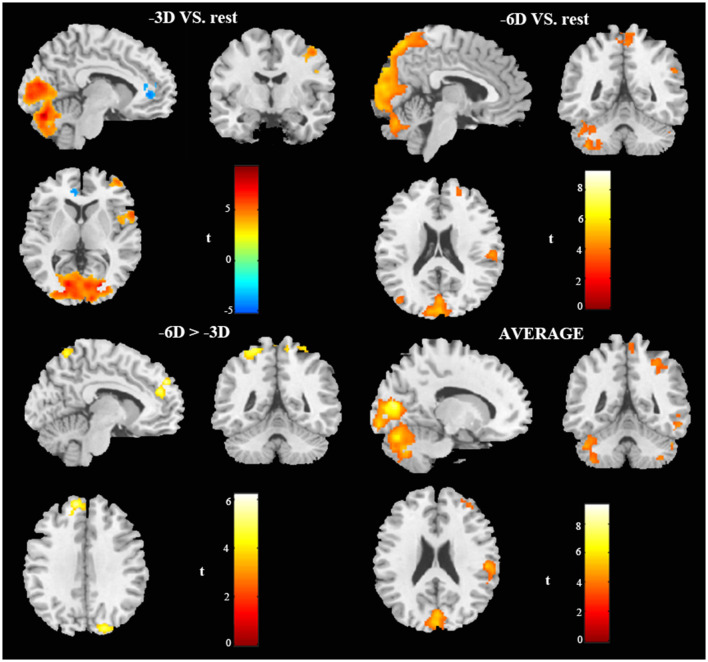
*In vivo* BOLD-functional magnetic resonance imaging (fMRI) visualization of cerebral activations during different conditions of ocular accommodation (projections of increased BOLD signals passing an family-wise error (FWE) cluster-level threshold of *P* ≤ 0.05). Regions activated shown in the sagittal view, coronal view, and cross-sectional view. The response magnitudes and MNI coordinates for the major brain areas based on the anatomical automatic labeling (AAL) template are listed in Table 1.

**Figure 3:**
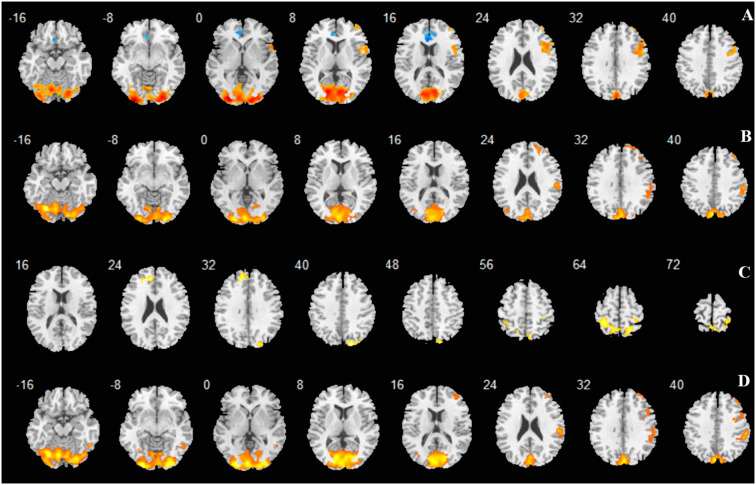
Regions activated shown as multi slices in a cross-sectional view. **(A)** -3D vs. rest; **(B)** -6D vs. rest; **(C)** -6D > -3D:−6D minus -3D; **(D)** Average: mean activation by -3D and -6D.

**Figure 4:**
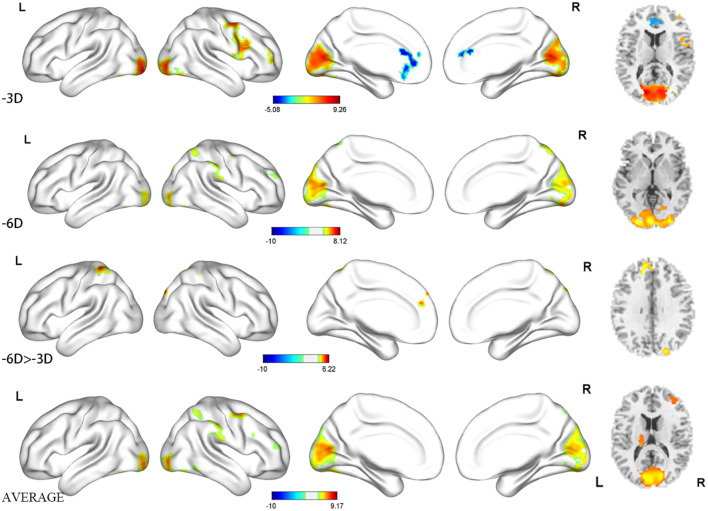
Regions activated shown as three-dimensional view and multi slices in a cross-sectional view.

The primary comparison of concern involved the contrast between the sustained steady-state accommodation condition (-3D accommodative demand) and the eye-closed rest condition [see Table 1 (-3D) and Figures 2, 3A, 4]. This contrast showed that the -3D task (simulating the state of normal eye use) recruited regions including the occipital lobe, cerebellum, precentral lobe, frontal lobe, and cingulate cortex. The occipital lobe was the most activated bilateral region. The subsections included were the lingual gyrus, cuneus, left calcarine fissure and surrounding cortex (Calcarine_L), and right calcarine fissure and surrounding cortex (Calcarine_R). The cerebellar activation areas included the left superior cerebellum (Cerebellum_Crus1_L and Cerebellum_6_L). Also, the foci of activation that occurred in the precentral lobe and frontal lobe were the right precentral lobe (Precentral_R) and the right inferior frontal gyrus, opercular part (Frontal_Inf_Oper_R) and right middle frontal cortex (Frontal_Mid_R). Of note, there was a significant suppression in the left anterior cingulate gyrus (Cingulum_Ant_L). This region was the only one that showed a negative response to the control condition, and this region did not show suppression in the other experimental condition with the -3D concave lens.

**Table 1:** Brain regions under different accommodative demand conditions.

	Brain region	Voxel size	MNI coordinate			Peak intensity
			*x*	*y*	*z*	
-3D	Occipital Lobe	6,187	−24	−90	−2	9.2563
	Cerebellum_Crus1_L	689	−6	−72	−34	9.2563
	Lingual Gyrus	1,446	−6	−75	−1	9.2563
	Cuneus	2,678	4	−81	23	9.2563
	Calcarine_L	1,246	−7	−79	6	9.2563
	Calcarine_R	1,002	15	−73	9	9.2563
	Cerebellum_6_L	864	−12	−63	−22	9.2563
	Cingulum_Ant_L	317	−6	42	1	−5.0818
	Precentral_R	554	52	2	31	6.1263
	Frontal_Inf_Oper_R	520	50	15	21	6.1263
	Frontal_Mid_R	242	42	54	4	5.0943
-6D	Occipital Lobe	6,589	−24	−90	−2	9.2677
	Cerebellum_Crus1_L	1,136	−6	−72	−34	9.2677
	Lingual Gyrus	1,654	−6	−75	−1	9.2677
	Parietal Lobe	1,557	50	−46	50	9.2677
	Cuneus	2,979	4	−81	23	9.2677
	Calcarine_L	1,257	−7	−79	6	9.2677
	Calcarine_R	904	15	−73	9	9.2677
	Cerebellum_6_L	1,098	−12	−63	−22	9.2677
	Precuneus_R	676	10	−56	44	9.2677
	Precuneus_L	557	−7	−56	48	9.2677
	Supramarginal_R	536	57	−32	34	5.4006
	Frontal_Mid_R	250	42	36	40	4.6621
-6D>-3D	Frontal_Sup_Medial_L	195	−14	42	30	5.7383
	Parietal Lobe	1,093	50	−46	50	6.223
	Precuneus_L	200	−7	−56	48	6.223
	Precuneus_R	198	10	−56	44	6.223
AVERAGE	Occipital Lobe	7,617	−24	−90	−2	9.304
	Cerebellum_Crus1_L	1,190	−6	−72	−34	9.304
	Lingual Gyrus	2,038	−6	−75	−1	9.304
	Cerebellum_6_L	1,184	−12	−63	−22	9.304
	Cuneus	3,143	4	−81	23	9.304
	Calcarine_L	1,393	−7	−79	6	9.304
	Calcarine_R	1,128	15	−73	9	9.304
	Frontal_Mid_R	356	40	48	28	4.8444
	Supramarginal_R	633	58	−28	40	5.2338
	Precentral_R	400	22	−21	71	6.9552
	Parietal_Inf_R	191	46	−46	50	5.0224
	Parietal_Sup_R	170	26	−60	62	5.0224

### Activation in -6D vs. Rest (Eye Closed) Conditions

The locations and peak intensities of the responses related to the contrast between the -6D (-6D effective accommodative demand) condition and the rest condition are listed in Table 1 (-6D) and Figures 2, 3B, 4.

The general pattern of the activated areas observed here was similar to the -3D vs. rest contrast, including the occipital lobe (Lingual gyrus, Cuneus, Calcarine_L, Calcarine_R), cerebellum (Cerebellum_Crus1_L, Cerebellum_6_L), and frontal lobe (Frontal_Mid_R). The activated voxel size in the occipital lobe was 6,589 cubic millimeters greater than 6,187 cubic millimeters in the previous -3D vs. rest contrast, and the peak intensity was 9.2677 also greater than 9.2563 in the previous contrast. The activated voxel size of the cerebellum (Cerebellum_Crus1_L and Cerebellum_6_L) respectively were 1,136 cubic millimeters and 1,098 cubic millimeters, greater than 689 cubic millimeters and 864 cubic millimeters in the previous -3D vs. rest contrast; The peak intensity of cerebellum was the same. Some other regions passed the significance threshold, including the parietal lobe, bilateral precuneus gyrus (Precuneus_R and Precuneus_L), and right supramarginal gyrus (Supramarginal_R). The precentral lobe and cingulate cortex failed to reach the threshold in the above comparison.

### Activation in -3D Minus -6D Conditions: More Activated by -3D Than -6D

When we compared the data from these two conditions, we found no brain region activated after cluster-level FWE correction.

### Activation in -6D Minus -3D Conditions: More Activated by -6D Than -3D

The contrast -6D (-6D effective accommodative demand) minus -3D [-3D effective accommodative demand; see Table 1 (-6D > -3D) and Figures 2, 3C, 4] highlighted the bilateral precuneus gyrus. Additionally, the left superior medial frontal gyrus (Frontal_Sup_Medial_L) and parietal lobe showed more activation.

### Activation in -3D Plus -6D Conditions: Mean Activation by -3D and -6D

Stimulation with the mean activation relative to -3D plus -6D conditions resulted in changes in the occipital lobe (Lingual gyrus, Cuneus, Calcarine_L, Calcarine_R), cerebellum (Cerebellum_Crus1_L, Cerebellum_6_L), and a right-hemisphere dominant circuit encompassing the frontal lobe (Frontal_Mid_R), precentral gyrus, and parietal lobe (Parietal_Inf_R, Supramarginal Gyrus, Parietal_Sup_R). Inspection of the results in Table 1 (AVERAGE) and the surface activation images in Figures 2, 3D, 4 demonstrate that the extent and intensity increases were discovered in the above brain areas.

## Discussion

The bottom-up pathway that causes the accommodative reflex is blurred visual signals → visual cortex → frontal eye field → median reticular structure near the pontine →oculomotor nucleus in midbrain reticular structure → Edinger-Westphal nucleus → ciliary ganglia → ciliary muscle (Agarwal et al., 2016; May et al., 2016, 2019). At present, there are few studies on the innervation above the E-W nucleus level. It is also known less about the top-down neural mechanisms of ocular accommodative function. For example, how the high-level cortex of the brain calculates and regulates accommodative signals from the eyes, how these brain signals enter the E-W nucleus, and regulate the preganglionic neurons in the E-W nucleus, and eventually transform into motor signals that govern the ciliary muscle.

We did not find non-accommodating visual perception as the control, even in the absence of light stimulation, there is tonic accommodation in the eyes (Chirre et al., 2016). In our study, we chose the eyes closed state as a control to compare it to the -3D and -6D accommodated open eyes. The functional activities may reflect two effects, one was the effect of accommodation, the other was the effect of open eyes, which was known that significantly changes the metabolic activity in the whole brain (Riedl et al., 2014; Mortensen et al., 2018). To maximize the functional activation caused by accommodative stimuli, we repeatedly told subjects to pay attention to the “E” target and keep the visual target clear during the trial task.

Compared with the rest condition, we found that the BOLD signals increased in the -3D stimulus and -6D stimulus task conditions in regions including the occipital lobe and cerebellum; decreased or disappeared in brain regions including the frontal lobe, cingulate gyrus, and precentral lobe; and revealed newly activated regions in the parietal lobe, precuneus lobe, and supramarginal gyrus. During the execution of monocular accommodation between the two different types of visual stimuli (distance and diopter), these activations reflected top-down processing of spatial error signals and refractive error signals that ultimately turned into motor signals controlling ciliary muscle contraction. The possible top-down routes processing these signals may be: Occipital Lobe and Cerebellum first receive and process accommodative stimulus signals; Frontal lobe, Parietal lobe, Cingulate cortex, and Precentral gyrus form a neural network. After adjusting the incoming processed accommodative signals, they send back and output neural signals through the subcortical nucleus.

Compared with previous research (Richter et al., 2004), we did not find activation of the temporal lobe passed the significance threshold of cluster-level FWE correction. *In Richter’s study*, regions activated by the near/far response included the temporal lobe, but in regions activated by visual fixation, there was no temporal lobe activation reported. Therefore, the temporal lobes may process stimulus signals related to changes in the distance (dynamic spatial error) and have nothing to do with signal processing of stimulus changes in fixation status (static spatial and refractive error).

### Occipital Lobe Involvement in Ocular Accommodation

The activation of the lingual gyrus, cuneus, and left/right calcarine during the -3D task and -6D task, in contrast to the rest condition, increased along with the increased accommodative demand. This suggested that these regions may have contributed to estimating the retinal blur signals. According to previous studies, it has been shown that the lingual gyrus probably plays a critical role involuntary saccadic eye movements (Petit et al., 1993), word and visual form discriminations (Gulyas et al., 1994; Halgren et al., 1994; Beason-Held et al., 1998; Mechelli et al., 2000), visual memory (Nakayama et al., 1994; Roland and Gulyas, 1995) and visual attention maintenance (Omori et al., 1999). The cuneus has been implicated in multisensory integration and cognitive processing (Parise et al., 2014). Vanni et al. (2001) indicated that the anteromedial cuneus was in a temporal position with respect to the primary visual cortex V1 to adjust the information being transferred to the extrastriate cortices. The calcarine is commonly involved in the earliest stages of processing in the visual system, producing a marked effect in visual imagery processing (Klein et al., 2000), and lesions of the calcarine cortex can induce hemianopia (Heller-Bettinger et al., 1976; Celesia et al., 1980). In the present study, the activation pattern was bilateral. Based on this study, we conclude that these brain regions are sensitive to retinal blur signals; however, we do not have measures of the sequential activation of the visual cortex, so we cannot obtain the details and information flow related to how the retinal refractive error was processed and transmitted.

### Cerebellum Involvement in Ocular Accommodation

There are two major activated regions in the cerebellum during the -3D task and the -6D task, relative to the rest condition, namely, the left cerebellum crus 1 and left cerebellum 6, in which the voxel size and peak intensity also increased with increasing accommodative demand. Single-unit anatomical techniques have demonstrated that direct nerve connections between the midbrain near-response region and the cerebellum exist in rhesus monkeys. In the cerebellum, there are cell activities related to lens accommodation (Bando and Toda, 1991; Gamlin and Clarke, 1995; Gamlin et al., 1996; Zhang and Gamlin, 1998). Richter et al. (2000, 2004, 2005) studied neuroanatomical correlates of the near response and also revealed several activated regions localized to the cerebellar cortex. Furthermore, the effects of cerebellar lesions can be manifested as difficulties in focusing on near or far targets (Kawasaki et al., 1993).

### Precentral Gyrus Involvement in Ocular Accommodation

The right precentral gyrus was positively activated only during the -3D task and not in the rest condition. Sato and Takamori (1998) reported on a patient who experienced lateral gaze paresis due to a lesion in the right precentral gyrus. A previous study had suggested that the human frontal eye field (FEF) is located in the middle frontal gyrus and the precentral gyrus (Sato and Takamori, 1998). Iacoboni et al. (1997) found that the right precentral gyrus merged oculomotor and somatomotor space coding in the human brain. In the -6D task, the right precentral gyrus did not pass the FWE correction threshold. It possibly is relevant to decline activation (Richter et al., 2005).

### Frontal and Parietal Lobe Involvement in Ocular Accommodation

The right frontal lobe showed activation in the -3D task vs. rest comparison. No BOLD signal increases occurred in the frontal lobe in the -6D task vs. rest comparison, except for the parietal lobe. Some activations in the right prefrontal and superior parietal cortex had been observed during sustained visual attention to sensory stimuli (Pardo et al., 1991). Gamlin et al. (1996) identified the FEF region as modulating or controlling ocular accommodation. Additionally, neurons in the parieto-occipital cortex related to ocular accommodation were discovered in cats and monkeys (Bando and Toda, 1991). In the human parieto-occipital cortex, an fMRI study revealed its preference for near viewing (Quinlan and Culham, 2007). There is also evidence from monkey neurophysiology that the parieto-occipital cortex shows a bias for encoding near space (Hadjidimitrakis et al., 2011, 2012; Breveglieri et al., 2012, 2014). In our experiment, the findings of the additional recruitment of the parietal lobules (bilateral precuneus gyrus) in the -3D task and the -6D task comparisons revealed that the parietal lobules mediated ocular accommodation and visuospatial processing (Horvath et al., 2019). As the main subdivision of the human inferior parietal lobule, the right supramarginal gyrus has activity linked to the abilities of sequence learning in eye movements and visual word recognition (Stoeckel et al., 2009; Burke et al., 2013).

### Cingulate Cortex Involvement in Ocular Accommodation

Notably, the left anterior cingulate gyrus was negatively activated in the contrast comparing -3D task vs. rest. There are two ways to explain this phenomenon. First is from the aspect of eye movement. The left anterior cingulate gyrus is necessary for sustained goal-oriented responses to discriminated stimuli (Degos et al., 1993).The anatomical location of the Cingulate eye field (CEF) was in Cingulate cortex/posterior aspect of anterior cingulate (Coiner et al., 2019), this area reliably shows activation during saccade and pursuit missions (Berman et al., 1999; Matsuda et al., 2004). Cingulum_Ant_L in our experiment shows suppression, which may be related to the suppression of eye movements. Second, from the aspect of mental processing, the left anterior cingulate gyrus is an important region involved in emotional and cognitive processing (Yoshimura et al., 2009; Liu et al., 2017). As an important part of the default network, activities are inhibited during cognitive task stimulation, and the degree of suppression increases with the difficulty of cognitive tasks (Buckner and DiNicola, 2019). Although there is no clear anatomical evidence indicating the relationship between Cingulum_Ant_L and ocular accommodative function, it is clear that ocular accommodative signals are regulated by advanced cognitive brain regions. No activation in this brain region has been found in previous studies (Richter et al., 2000, 2004, 2005) about ocular accommodative stimuli task.

## Conclusion

In the present study, we conclude that (i) neural circuits were selectively activated during the -3D/-6D accommodative response to blur cues. The bilateral occipital lobes compute the retinal blur error signals from a distance and refractive targets; and (ii) a system involving cognitive-perceptual processing is engaged within the accommodative system.

## Data Availability Statement

All datasets generated for this study are included in the article.

## Ethics Statement

The studies involving human participants were reviewed and approved by Shuguang Affiliated Hospital of Shanghai University of Traditional Chinese Medicine Human Research Ethics Committee. The patients/participants provided their written informed consent to participate in this study.

## Author Contributions

XL, YC, and WT: article writing, implementation, and design of the trial. YY, HZ, and YS: participation in the design of the experiment. SZ, JT, and WM: planning and guidance for participating in the experiment.

## Conflict of Interest

The authors declare that the research was conducted in the absence of any commercial or financial relationships that could be construed as a potential conflict of interest.

## Footnotes

^1^http://www.fil.ion.ucl.ac.uk/spm/software/spm12/

^2^https://www.mathworks.com/

## Abbreviations

SPAM: statistical parametric anatomical mapping
BOLD-fMRI: blood oxygenation level-dependent functional magnetic resonance imaging
MPRAGE: magnetization-prepared rapid gradient-echo imaging
EPI: echo-planar imaging
D: diopter
AAL: anatomical automatic labeling
MNI: Montreal Neurological Institute.
